# Quantitative Profiling of Oxylipins in Acute Experimental Intracerebral Hemorrhage

**DOI:** 10.3389/fnins.2020.00777

**Published:** 2020-09-23

**Authors:** Jun-Jie Yuan, Qiong Chen, Xiao-Yi Xiong, Qin Zhang, Qi Xie, Jia-Cheng Huang, Guo-Qiang Yang, Chang-Xiong Gong, Zhong-Ming Qiu, Hong-Fei Sang, Wen-Jie Zi, Qian He, Rui Xu, Qing-Wu Yang

**Affiliations:** Department of Neurology, Xinqiao Hospital, The Army Medical University (Third Military Medical University), Chongqing, China

**Keywords:** oxylipin, intracerebral hemorrhage, cyclooxygenase, lipoxygenase, cytochrome P450

## Abstract

Oxylipins are a series of bioactive lipid metabolites derived from polyunsaturated fatty acids that are involved in cerebral homeostasis and the development of intracerebral hemorrhage (ICH). However, comprehensive quantification of the oxylipin profile in ICH remains unknown. Therefore, an ICH mouse model was constructed and liquid chromatography tandem mass spectrometry was then performed to quantify the change in oxylipins in ICH. The expression of the oxylipin relative enzymes was also reanalyzed based on RNA-seq data from our constructed ICH dataset. A total of 58 oxylipins were quantifiable and the levels of 17 oxylipins increased while none decreased significantly in the first 3 days following ICH. The most commonly increased oxylipins in ICH were derived from AA (10/17) and EPA (4/17) followed by LA (2/17) and DHA (1/17). 18-HEPE from EPA was the only oxylipin that remained significantly increased from 0.5 to 3 days following ICH. Furthermore, 14 of the increased oxylipins reached a peak level on the first day of ICH, and soon decreased while five oxylipins (PGJ2, 15-oxo-ETE, 12-HEPE, 18-HEPE, and 5-oxo-ETE) had increased 3 days after ICH suggesting that the profile shifted with the progression of ICH. In our constructed RNA-seq dataset based on ICH rats, 90 oxylipin relative molecules were detected except for COX. Among these, *Cyp4f18, Cyp1b1, Cyp2d3, Cyp2e1, Cyp1a1, ALOX5AP*, and *PLA2g4a* were found up-regulated and *Cyp26b1* was found to decrease in ICH. In addition, there was no significant change in *sEH* in ICH. This study provides fundamental data on the profile of oxylipins and their enzymes in ICH. We found that the profile shifted as the progression of ICH and the metabolism of arachidonic acid and eicosapentaenoic acid was highly affected in ICH, which will help further studies explore the functions of oxylipins in ICH.

## Introduction

Oxylipins are polyunsaturated fatty acid (PUFAs) oxidation products formed via one or more mono- or dioxygen-dependent reactions (Gabbs et al., 2015). These PUFAs consist of arachidonic acid (AA), docosahexaenoic acid (DHA), eicosapentaenoic acid (EPA), linoleic acid (LA), and α-linolenic acid (ALA). Once liberated by cytosolic phospholipase A2 (cPLA2) from membrane phospholipids, free PUFAs can be metabolized to form oxylipins via auto-oxidation or enzymes including cyclooxygenase (COX), lipoxygenase (LOX), and cytochrome P450 (CYP)-soluble epoxide hydrolase (sEH) pathways (Caligiuri et al., 2017). Given their short half-time and low content, oxylipins are found all over the tissues and support the body’s homeostasis by regulating many important physiological processes such as inflammation, blood coagulation, and vascular function (Nayeem, 2018).

Arachidonic acid and DHA are the major PUFAs in the brain, nevertheless the profile of oxylipins does not completely reflect the constitutions of PUFAs, but also depends on the oxygenases available and the oxygenases’ affinity for a specific substrate PUFAs (Gabbs et al., 2015). Nearly half of the oxylipins in the brain are derived from AA, while less than 20% of oxylipins are produced from DHA which accounts for 40% of total PUFAs (Ferdouse et al., 2019). Thus comprehensive analysis of the oxylipin profile is necessary to clarify the constitutions and possible functions of oxylipins in the brain. There is also research focusing on some oxylipins or oxylipin related enzymes such as COX and 5/12/15-LOX which in acute experimental ICH model indicates that oxylipins are important regulatory factors of ICH (Wu et al., 2011; Rosso et al., 2014; Xu et al., 2017; Karuppagounder et al., 2018; Han et al., 2019). However, the specific profile of oxylipins and the overall expression pattern of their relative enzymes in ICH has not been fully discovered yet. On the other hand, different from ischemia stroke which shows masses of PUFAs released from brain parenchyma, erythrocytes entering the brain during ICH will be gradually lysed releasing substantial cell membrane for further metabolism. The constitution of PUFAs in blood is quite different with the brain, which may specifically affect the metabolism of certain oxylipins in ICH.

In order to comprehensively quantify the profile of oxylipins in ICH, we constructed an ICH mouse model using autologous blood injection and the tissues around hematoma were collected in the acute phase of ICH. The concentrations of oxylipins were then detected via liquid chromatography tandem mass spectrometry (LC/MS). On the other hand, the mRNA levels of oxylipin relative enzymes were also reanalyzed based on our previously constructed RNA-sequence (RNA-seq) library of ICH. We found that the profile of oxylipins shifted as the progression of ICH and the metabolism of AA and EPA was highly affected in ICH. This study extends our understanding of the change in oxylipin profile and their enzymes in ICH, which will help explore the functions of oxylipins in ICH.

## Materials and Methods

### Animals

**FIGURE 1:**
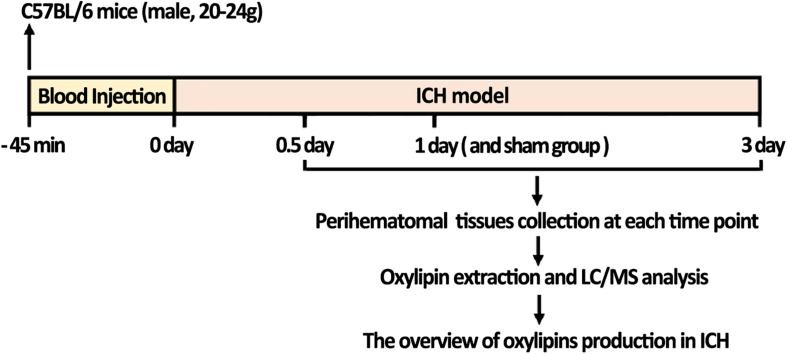
The flow chart of the study; ICH mice were constructed and collected 0.5, 1, and 3 days after ICH. LC-MS/MS analysis was then performed to quantify the change of oxylipins in the brain. Mice injected with saline and collected after 1 day were used as the sham control.

As showed in the flow chart of the present study, a total of 40 mice were used for oxylipins detection (Figure 1). C57BL/6 mice (male, 8-week-old, 20-24g) were purchased from the Animal Center of the Army Medical University (Chongqing, China). The mice were raised in the specific pathogen-free grade environment with 12 h light/12 h dark cycle and given ad libitum access to food and water. All procedures and animal experiments were approved and conducted in accordance with the Animal Ethics Committee of the Army Medical University.

### ICH Model

The ICH model was conducted according to our previously developed method (Yuan et al., 2019). Briefly, mice were immobilized on the stereotaxic apparatus (RWD Life Science Co, Shenzhen, China) after being anesthetized with 3% isoflurane for induction and 1.5% for maintenance. Whole blood (20 μl) without anticoagulant was obtained from its tail and then injected into the left striatum (0.8 mm anterior and 2 mm lateral of bregma, at a depth of 3.5 mm) using a syringe pump (Hamilton, Bonaduz, AG) at 2.0 μl/min. The microinjector was detained for 10 min. The sham group was injected with 20 μl saline using the same procedures. Unsuccessful ICH models that were asymptomatic or dead were excluded from this study. Perihematomal cerebral tissues were collected for oxylipins quantification at 0.5, 1, and 3 days after ICH or 1 day after saline injection.

### Oxylipin Extraction

Oxylipin extraction was performed according to the previously developed method (Ostermann et al., 2015; Sun et al., 2019). Ultra-performance liquid chromatography (UPLC)-grade methanol, acetonitrile, acetic acid, isopropanol, hydrochloric acid (HCl), menthyl formate, n-hexane, and water were purchased from Merck (Darmstadt, GER) and the standard samples of all oxylipins were purchased from Cayman (Michigan, United States). Every 100 mg of brain tissue was homogenized with 2ml of 4°C methanol solution. After 5 min of vortex, the sample was laced for protein precipitation at low temperature. Twenty microliter of 1 μM internal standard mixture was added to each sample and centrifuged at 5000 rpm for 10 min. Supernatants were taken and dried within nitrogen. Eight milliliter of 10% methanol water was used as a dissolvent and the pH (3.5) was adjusted using 0.2 mol/l HCl. The solid phase extraction column (SepPak tC18 SPE, 6 ml, 500 mg, 37–55 μm particle, waters) was equilibrated by 5 ml methanol followed by 5 ml water. Samples with appropriate pH were then transferred to the prepared solid phase extraction column. The column was rinsed with 4 ml water followed by 5 ml n-hexane. Elution was performed using 10ml menthyl formate and the eluates were collected. Eluates were blown dry with nitrogen, then reconstituted with 200 μl methanol-water (v:v = 1:1) for 30 s, and the supernatants were then available for oxylipins analysis.

### Liquid Chromatography and Mass Spectrometry

Liquid chromatography tandem mass spectrometry was performed according to the previously developed method (Willenberg et al., 2015). Briefly, the sample extracts were analyzed using an LC-ESI-MS/MS system (UPLC, Shim-pack UFLC SHIMADZU CBM A system, https://www.shimadzu.com/; MS, QTRAP^®^ 6500+ System, https://sciex.com/). The analytical conditions were as follows, UPLC: column, ACQUITY UPLC HSS T3 (1.8 μm, 2.1 mm × 100 mm); solvent system, A: acetonitrile/water (60/40V,0.02% acetic acid), B: acetonitrile / isopropanol (50/50 V); gradient program, A/B (99.9:0.1 V/V) at 0 min, 70:30 V/V at 2.0 min, 50:50 V/V at 4 min, 1:99 V/V at 5.5 min, 1:99 V/V at 6.0 min; 99.9:0.1 V/V at 7.0 min; stop at 10.0 min; flow rate: 0.40 ml/min; temperature: 40°C; injection volume: 10 μl. The effluent was alternatively connected to an ESI-triple quadrupole-linear ion trap (QTRAP)-MS. LIT and triple quadrupole (QQQ) scans were acquired on a triple quadrupole-linear ion trap mass spectrometer (QTRAP), QTRAP^®^ 6500+ LC-MS/MS System, equipped with an ESI Turbo Ion-Spray interface, operating in negative ion mode and controlled by Analyst 1.6.3 software (Sciex). The ESI source operation parameters were as follows: ion source, turbo spray; source temperature 550°C; Multiple reaction monitoring (MRM) scan, negative ion mode; EPI scan range: *m*/*z*: 50–640; scan speed: 1000 da/s; curtain gas, ion source gas 1 and ion source gas 2 were delivered at 35, 40, and 40 psi, respectively. These jobs were completed with the help of the Wuhan Metware Biotechnology Co. Ltd. (Wuhan, China).

### Statistical Analysis

Unsupervised principal component analysis (PCA) was performed by R. The cluster analysis results of metabolites were presented as heatmaps. Differential analysis was performed to compare the content of the 0.5, 1, and 3 days ICH groups to the sham group using Orthogonal projections to latent structures (OPLS-DA) by metaboanalyst software and the Mann-Whitney test. Variable importance in projection (VIP) values were extracted from OPLS-DA results, which also contained score plots and permutation plots, generated using the R package. Significantly produced metabolites between groups were determined by VIP ≥ 1, *p* < 0.05 using the Mann–Whitney U test and absolute Log_2_FC ≥ 1 or ≤0.5 (Sun et al., 2019). The identified metabolites were annotated using the Kyoto Encyclopedia of Genes and Genomes (KEGG) database, and the annotated metabolites were then mapped to the KEGG pathway database. Pathways with significantly produced metabolites were then fed into metabolite sets enrichment analysis, the significance of which was determined by the *p*-value.

### RNA-Seq Dataset Processing

The RNA-seq dataset was previously constructed and based on 13-month and 22-month-old SD rats using the contralateral hemisphere as control at 3 days after ICH. To analyze the expression of oxylipins relative enzymes in ICH, all of the cPLA2, COX, LOX, CYP, and sEH were first selected. Their raw DNA reads were first discarded if there were more than 2-N base reads, clipping adaptor, low quality bases (less than 20), and too short (less than 16 nt) using the Cutadapt software according to a previous study (Kechin et al., 2017). After that, the reads were compared to the Rattus norvegicus genome sequences (Rnor 6.0) and annotation file (Rnor 6.0.82) from the Ensembl database^1^. After, the reads were aligned to the rat genome using TopHat2 according to a previous study with an end-to-end method allowing two mismatches (Kim et al., 2013). The aligned DNA reads with more than one genomic localization were also discarded and the uniquely localized reads were calculated for the read number and RPKM value (The reads per kilobase and per million). We then obtained the gene coverage and depth, read distribution around transcription starting sites, and transcription terminal sites. After attaining the level of gene expression in all samples, we also obtained the differentially expressed genes (DEGs) using edgeR, one of the R packages, according to a previous study which compared the adjacent time point samples to investigate the genes that were regulated by aging or by comparing the treated and normal ICH samples at the same time points (Robinson et al., 2010). For each gene, we also obtained the p-value according to the model of the negative binomial distribution and estimated the fold change using this package when *p*-value < 0.01 and 2-fold changes (Log_2_FC > 1) were reached.

## Results

### Oxylipin Profile in the Acute Phase of Intracerebral Hemorrhage

**FIGURE 2:**
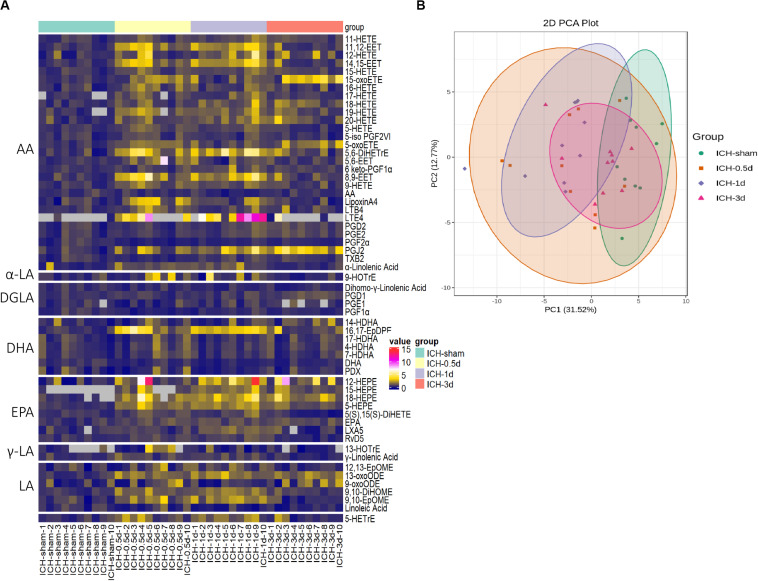
The profile of oxylipins in the acute phase of ICH. **(A)** The hierarchical cluster of the heat map shows the profile of oxylipins in ICH (*n* = 10). **(B)** Principal component analysis (PCA) of the oxylipins in four groups (*n* = 10).

To gain better insight into the full spectrum of oxylipins alterations initiated by ICH at the molecular level, LC/MS was performed on the brain samples obtained from mice sacrificed at 0.5, 1, and 3 days following ICH and 1 day after saline injection for the sham group (Figure 1). In this study, 58 oxylipins were quantifiable in the brain (Figure 2A and Supplementary Table S1). Consistent with other studies on the profile of cerebral oxylipins, nearly half of oxylipins were derived from AA (48.3%). EPA derived oxylipins ranked second (13.8%), followed by DHA (12.1%), LA (10.3%), DGLA (8.6%), α-LA (3.5%), and γ-LA (3.5%) (Figure 2A). Based on the assessment of PCA, the profile of oxylipins in ICH was shifted compared to the sham group, and the differences in 1 day seemed to be the biggest, while the profile at 3 days was largely overlapped with the sham group (Figure 2B). Further OPLS-DA analysis showed that the levels of 17 oxylipins were increased and none were significantly decreased in ICH (Table 1). Most of the increased oxylipins in ICH were derived from AA (10/17) and EPA (4/17), followed by LA (13-oxoODE; 9,10-EpOME) and DHA (16,17-EpDPE) (Table 1). With respect to oxylipins’ patterns, 4 out of 8 oxylipins from EPA and 10 out of 28 oxylipins from AA were found increased in the acute phase of ICH indicating that the metabolism of AA and EPA was highly affected in ICH (Figure 2A).

**TABLE 1:** Up-regulated oxylipins in the acute phase of ICH.

**Oxylipin**	**Sham**	**ICH 0.5 day**				**ICH 1 day**				**ICH 3 days**			
	**Content (nmol/g)**	**Content (nmol/g)**	**VIP**	***p*-value**	**log_2_ FC**	**Content (nmol/g)**	**VIP**	***p*-value**	**log_2_ FC**	**Content (nmol/g)**	**VIP**	***p*-value**	**log_2_ FC**
**AA**													
LTE4	0.022 ± 0.011	0.104 ± 0.049	0.97	N/A	2.24	0.155 ± 0.089*	1.41	N/A	2.81	0.053 ± 0.069	0.32	N/A	1.27
5,6-DiHETrE	0.004 ± 0.002	0.010 ± 0.005*	1.44	0.00	1.48	0.013 ± 0.005*	1.46	0.00	1.84	0.006 ± 0.003	0.99	0.03	0.71
8,9-EET	0.021 ± 0.007	0.062 ± 0.032*	1.51	0.00	1.56	0.062 ± 0.023*	1.55	0.00	1.56	0.030 ± 0.013	1.11	0.08	0.51
14,15-EET	0.071 ± 0.018	0.171 ± 0.063*	1.50	0.00	1.26	0.204 ± 0.051*	1.60	0.00	1.51	0.094 ± 0.048	0.70	0.19	0.39
11,12-EET	0.054 ± 0.013	0.133 ± 0.048*	1.53	0.00	1.30	0.148 ± 0.035*	1.59	0.00	1.45	0.070 ± 0.033	0.75	0.19	0.37
PGJ2	1.159 ± 0.458	2.257 ± 1.039	1.01	0.01	0.96	3.033 ± 1.343*	1.31	0.00	1.39	3.845 ± 1.086*	1.91	0.00	1.73
12-HETE	3.685 ± 2.032	6.344 ± 4.513	0.99	0.11	0.78	8.100 ± 5.020*	1.22	0.02	1.14	6.100 ± 3.300	1.12	0.07	0.73
LipoxinA4	0.003 ± 0.001	0.006 ± 0.003*	1.11	0.00	1.25	0.004 ± 0.002	0.74	0.04	0.66	0.003 ± 0.001	0.04	0.85	0.05
15-oxoETE	0.048 ± 0.011	0.096 ± 0.033	1.30	0.00	0.99	0.071 ± 0.035	0.62	0.07	0.56	0.148 ± 0.061*	1.73	0.00	1.61
5-oxoETE	0.101 ± 0.024	0.184 ± 0.033	1.53	0.00	0.87	0.140 ± 0.044	0.86	0.03	0.48	0.209 ± 0.078*	1.68	0.00	1.06
**EPA**													
12-HEPE	0.079 ± 0.061	0.256 ± 0.303	1.20	0.10	1.69	0.308 ± 0.290*	1.37	0.04	1.96	0.236 ± 0.193*	1.39	0.03	1.58
18-HEPE	0.004 ± 0.001	0.012 ± 0.006*	1.00	0.01	1.46	0.012 ± 0.005*	1.24	0.00	1.52	0.010 ± 0.005*	1.62	0.00	1.30
5-HEPE	0.005 ± 0.001	0.011 ± 0.003*	1.67	0.00	1.09	0.012 ± 0.002*	1.64	0.00	1.18	0.008 ± 0.002	1.69	0.00	0.63
15-HEPE	0.003^*a*^	0.005 ± 0.002	1.20	N/A	0.88	0.005 ± 0.001*	1.57	N/A	1.01	0.004 ± 0.002	2.04	N/A	0.70
**DHA**													
16,17-EpDPE	0.030 ± 0.008	0.092 ± 0.046*	1.48	0.00	1.62	0.098 ± 0.012*	1.63	0.00	1.71	0.048 ± 0.028	1.06	0.07	0.68
**LA**													
13-oxoODE	0.010 ± 0.003	0.018 ± 0.004	1.48	0.00	0.96	0.020 ± 0.005*	1.40	0.00	1.11	0.019 ± 0.004	1.72	0.00	0.98
9,10-EpOME	0.004 ± 0.002	0.009 ± 0.003	1.16	0.00	0.98	0.010 ± 0.003*	1.23	0.00	1.21	0.004 ± 0.002	0.01	0.84	−0.06

Data present mean ± SD. ^*a*^The content in some samples was below the detection limit leading to an error on SD calculation. *vs Sham group, VIP ≥ 1, *p* < 0.05 using the Mann–Whitney U test and Log_2_FC ≥ 1. VIP values were calculated by an OPLS-DA model using the metaboanalyst software.

**FIGURE 3:**
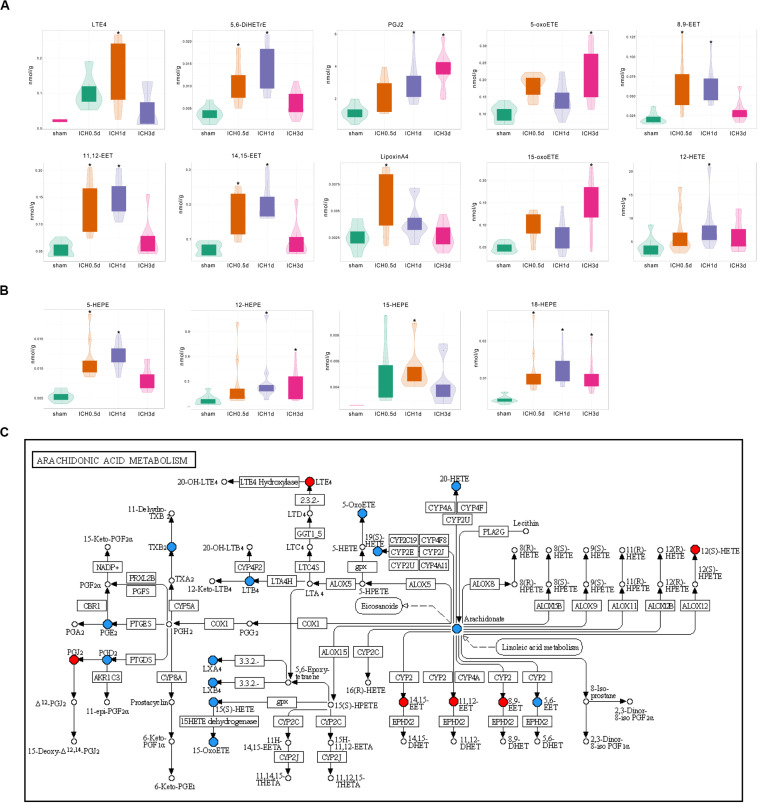
Differential production of oxylpins in ICH. **(A)** Violin plots show the increase in oxylipins from AA in ICH (* vs Sham group, VIP ≥ 1, *p* < 0.05 using the Mann–Whitney U test and Log_2_FC ≥ 1, *n* = 10); **(B)** Violin plots show the increase in oxylipins from EPA in ICH (* vs Sham group, VIP ≥ 1 *p* < 0.05 using the Mann–Whitney U test and Log_2_FC ≥ 1, *n* = 10); **(C)** Kyoto Encyclopedia of Genes and Genomes (KEGG) pathway analysis of the identified differential oxylipins from AA at 1 day after ICH. Red indicates a significant increase in oxylipin; blue indicates that the oxylipin was detected but did not change significantly compared to the sham group and white indicates that the oxylipin was not quantitative in the brain.

Among the increased oxylipins in ICH, LTE4, 5,6-DiHETrE, PGJ2, 5-oxoETE, and 8,9-EET were the top five metabolites that increased most from AA in ICH (Figure 3A); 12-HETE, an important effector of AA, also showed an increased trend in ICH and was significant increased 1 day following ICH (Figure 3A); 18-HEPE belonging to EPA was increased throughout the whole acute phase of ICH (Figure 3B); 16,17-EpDPE, the only increased DHA derived oxylipin returned to normal 3 days after ICH (Supplementary Figure S1A).

Furthermore, the profile of oxylipins shifted with the progression of ICH. Eight of 17 increased oxylipins had already increased 0.5 day after the injection of blood and kept increasing to 14 after 1 day, but these oxylipins went back to normal rapidly with only 5 metabolites that were increased 3 days after ICH, which matched the trends seen in PCA (Table 1). Defined by the fold change, 12-HEPE, 16,17-EpDPE, 8,9-EET, 5,6-DiHETrE, and 18-HEPE were the top five metabolites that increased most in the ICH 0.5 day group and they kept increasing 1 day after ICH with LTE4 ranked first (Table 1). However, PGJ2, 15-oxo-ETE, 12-HEPE, 18-HEPE, and 5-oxo-ETE were the only oxylipins that increased 3 day after ICH suggesting that the profile of oxylipin at 3 days after ICH was different with the former (Table 1).

Further KEGG analysis provided clues regarding the causes of changes in the oxylipin profile. The KEGG pathway of AA metabolism at 0.5 and 1 days after ICH revealed that most of the increased oxylipins were produced by CYP and LOX (Figure 3C and Supplementary Figure S1B). Oxylipins produced by COX were more predominant 3 days after ICH, suggesting that the activation of COX might be slower than the CYP and LOX pathways (Supplementary Figure S1C). Other oxylipins also showed similar characteristics. Unlike LOX that was activated through the whole experiment, most products of CYP including 16,17-EpDPE from DHA and 9,10-EpOME from LA returned to normal at 3 days after ICH. These results might hint that the profile of oxylipins in ICH might relate to the activation and selectivity of their enzymes.

### The Expression of Oxylipin Relative Enzymes in ICH

**FIGURE 4:**
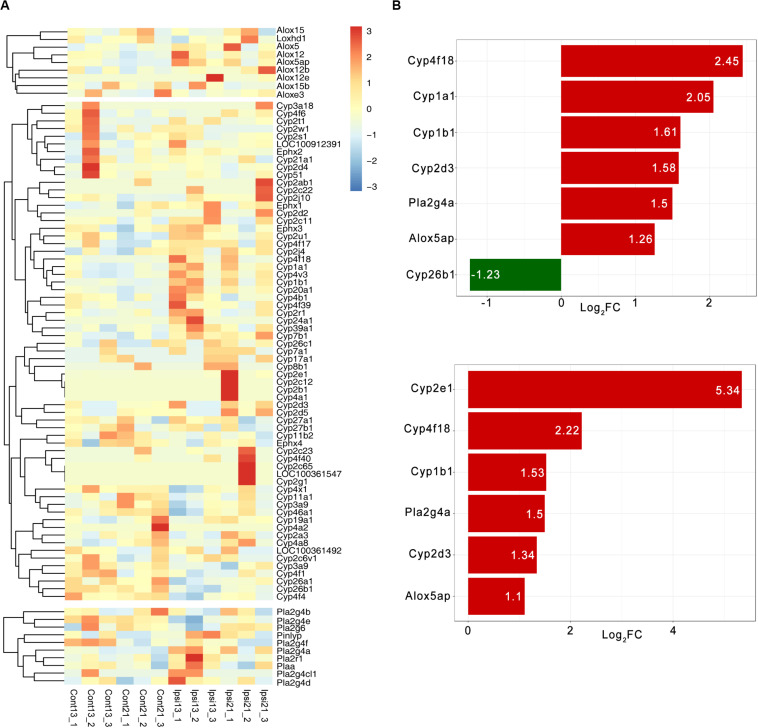
The expression pattern of oxylipins relative enzymes in ICH. **(A)** The hierarchical cluster of the heat map shows the expression of oxylipins related enzymes in ICH (*n* = 3); **(B)** Differential expression of oxylipins related enzymes in 13-month-old ICH rats **(top panel)** and 22-month-old ICH rats **(bottom panel)** (vs contralateral hemisphere, *p* < 0.01 and Log_2_FC > 1, *n* = 3).

The RNA-seq dataset was previously constructed and was based on 13-month and 22-month-old SD rats at 3 days after ICH, which corresponded with the common age of ICH. A total of 90 oxylipin relative molecules were quantifiable in the brain except for COX (Figure 4A). The expression patterns of oxylipins relative enzymes were extremely similar in both the 13-month and 22-month-old ICH group and CYP was the most affected by ICH (Figure 4A). Among these, *Cyp4f18, Cyp1b1*, and *Cyp2d3* was found up-regulated in both the 13-month and 22-month groups after ICH, except that *Cyp2e1* was mainly increased in the 22-month group and *Cyp1a1* was mainly increased in the younger group (Figure 4B). *Cyp26b1* was found to have a decreased trend in ICH and was more pronounced in the younger group (Figure 4B). In the cluster of LOX, *ALOX5AP* was showed to have two times up-regulated and *PLA2g4a* was the only cPLA2 that kept increased in ICH (Figure 4B). In addition, there was no significant change of *sEH* in ICH (Figure 4A).

## Discussion

This study provides foundational data concerning on the alterations of oxylipins profile in ICH by quantitative LC/MS. Fifty-eight oxylipins were quantifiable in the study and nearly half of oxylipins were derived from AA in either the sham or ICH group, which was similar with the feature of cerebral oxylipins (Ferdouse et al., 2019). Different to other research, EPA derived oxylipins comprised 13.8% of the profile of cerebral oxylipins, which was a slightly larger proportion than that accounted for by DHA-derived oxylipins. The disparity might be caused by the differences in databases where some DHA derived oxylipins were missed during LC/MS analysis. Hitherto, there is still no standard oxylipins database being published and the biggest database about cerebral oxylipins was constructed by *Harold M. Aukema*, which gauged 87 oxylipins in the brain (Ferdouse et al., 2019). Given that most of the content of oxylipins are extremely low in the tissues, methods with higher accuracy and sensitivity are urgently needed.

OPLS-DA analysis showed that 17 out of 58 oxylipins were significantly increased and none were decreased in ICH. Among the quantifiable oxylipins from EPA and AA in the brain, 4/8 and 10/28 were increased in ICH, respectively, which was higher than other clusters of oxylipins. In this respect, the metabolism of EPA and AA was mostly affected in ICH. However, it is interesting to note that the brain maintains very low levels of EPA but contains high levels of DHA (Chen et al., 2013). Besides, masses of oxylipins are quickly produced and peaked after the first day of ICH. These up-regulated oxylipins return to normal rapidly except for 12-HEPE and 18-HEPE, while the five oxylipins that increased 3 days following ICH indicate that the profile of oxylipins shifted with the progression of ICH. Interestingly, the regular pattern of oxylipins corresponded with the lysis of RBCs in hematoma. The lysis of RBCs relies on the phagocytosis of microglia/macrophages in parenchyma and compared to 1 day after ICH, most of the RBCs in hematoma are absorbed as the activation and infiltration of microglia/macrophages occurs 3–7 days following ICH (Fang et al., 2014; Zhao et al., 2015; Wang et al., 2017, 2018) 2018). Therefore, the lysis of RBCs may cause more dramatic effects on the production of oxylipins with the progression of ICH until the hematoma is completely absorbed. For these reasons, we infer that the phospholipids from RBCs of hematoma are another source for the production of oxylipins in the acute phase of ICH, especially those derived from EPA. To further prove this hypothesis, it is nececessary to compare the increase in brain lipids to the fatty acid and oxylipin levels in blood, and metabolic flux isotope analysis is warranted in the future.

The functions of oxylipins involve many aspects of body homeostasis including innate immunity, inflammation, cardiac function, blood coagulation, and vascular tone regulation (Nayeem, 2018). Compared to oxylipins derived from n–3 PUFAs (DHA, EPA), those derived from n–6 PUFAs (AA, LA) generally, but not always, exhibit pro-inflammatory, vasoconstrictive, and proliferative effects (Gabbs et al., 2015). In the acute phase of ICH, the increased production of LTE4, PGJ2, 5-oxo-ETE, and 15-oxo-ETE derived from AA may exhibit vasoconstrictive, pro-apoptotic, pro-inflammatory, and chemoattractant effects which are inclined to aggregate the damage of ICH, while 5,6-DiHETrE, 12-HETE, and EETs show opposing effects such as vasodilative, anti-apoptotic, or anti-inflammatory activities (Sudhahar et al., 2010; Bennett and Gilroy, 2016; Figueiredo-Pereira et al., 2016; Shearer and Walker, 2018; Rahman et al., 2019; Wójcik et al., 2020). Of note, these oxylipins of AA change with the progression of ICH and the destructive oxylipins appear to peak 3 days after ICH when the protective oxylipins of AA have descended suggesting that AA-derived oxylipins tend to cause damage with the development of ICH. Studies show that HEPE tends to present anti-inflammatory effects and 12-HEPE can improve glucose metabolism by promoting glucose uptake through the insulin-like intracellular signaling pathway (Leiria et al., 2019). 18-HEPE, the precursor of the E-series specialized pro-resolving mediators, is another inhibitor of inflammation, and can halt the development of maladaptive cardiac remodeling and atherosclerosis (Endo et al., 2014; Liu et al., 2018). 16,17-EpDPE derived from DHA is a vasodilator which regulates the calcium-potassium (BK) channel in arterial smooth muscle cells and correlates with the decrease of white matter hyperintensity in older populations with a high risk of dementia (Wang et al., 2011; Shinto et al., 2020). These properties of EPA- and DHA-derived oxylipins suggest that they are potential defenders in ICH. However, direct evidence is urgently needed to explore the functions of up-regulated oxylipins in ICH.

There is still no research providing full information on the expression pattern of oxylipin relative enzymes in ICH, thus transcriptome studies related to ICH such as RNA-seq and microarray can be an alternative source. Nevertheless, only a few studies contain such information and the profiles of oxylipins relative enzymes in each study are different (Carmichael et al., 2008; Rosell et al., 2011; Sang et al., 2017; Walsh et al., 2019). Some researchers have also used peripheral blood mononuclear cells as a source of mRNA for gene expression profile analysis instead of perihematomal tissues, but these studies do provide important clues on the change in oxylipin relative enzymes in ICH (Sang et al., 2017; Walsh et al., 2019). Substantial peripheral inflammatory cells will infiltrate into the brain through the breakdown of the blood–brain barrier (BBB) and are involved in the ICH-induced inflammatory response (Xiong and Yang, 2015). These inflammatory cells that are full of oxylipin relative enzymes are involved in the lysis of RBCs and thus may be closely linked to the change of oxylipins in ICH. Consistent with our results, ALOX5AP is found to increase in the acute phase of ICH (Rosell et al., 2011; Walsh et al., 2019). Besides, there are two studies, whose samples are taken during 3h and 24h after the onset of ICH, showing that ALOX5AP and Cyp1b1 is significant increased in this very early stage of ICH (Carmichael et al., 2008; Sang et al., 2017). This may hint that the up-regulation of ALOX5AP and Cyp1b1 can sustain through the acute phase of ICH.

ALOX5AP is associated with the development of ICH, but the effect of ALOX5AP on ICH is as yet undiscovered (Kim et al., 2011). ALOX5AP is closely linked with ALOX5 and they are both required for leukotriene synthesis. Their metabolites include LTE4 and 5-oxo-ETE which exhibits pro-apoptotic or pro-inflammatory effects and targeting ALOX5 can effectively reduce ferroptosis and improve outcomes following ICH, which makes us wondering if ALOX5AP could regulate the damage of ICH (Postula et al., 2016; Karuppagounder et al., 2018). Other detected enzymes may also be linked to the over production of oxylipins in ICH. For example, Cyp2 is inclined to participate in DHA metabolism and may be responsible for the production of 16,17-EpDPE (Wang et al., 2011). Cyp4 is the putative metabolic enzyme of 18-HEPE and ALOX12, up-regulated in the early stage of ICH, is responsible for 12-HEPE production (Kalsotra et al., 2004). However, there remain many oxylipins, such as 5,6-DiHETrE that are not directily linked with given enzymes, so future studies are needed to verify the relationship between metabolite enzymes and oxylipins. An important limitation of the RNA-seq data is the mismatch between the model used for oxylipins and the RNA-seq. The 13-month and 22-month-old SD rats were selected because age is an independent risk factor for ICH and most ICH happens in older populations. The 13-month and 22-month-old rats may better reflect the real situation of ICH but create certain biases in understanding of the relationship between oxylipins and their relative enzymes.

Despite these limitations, this is the first study to have comprehensively assessed the changes of oxylipins and their relative enzymes in ICH. The profile of oxylipins shifted with the progression of ICH and the metabolism of AA and EPA was highly affected in ICH. A few specific oxylipins have been discovered in ICH, which are worth exploring in future studies to identify the functions and regulatory mechanisms and to determine whether these oxylipins can be potential targets for the treatment of ICH.

## Data Availability Statement

The RNA-seq dataset was uploaded at Gene Expression Omnibus (GEO) with accession number GSE149317.

## Ethics Statement

The animal study was reviewed and approved by Animal Ethics Committee of the Army Medical University.

## Author Contributions

J-JY and Q-WY conceptualized this study and designed the experiments. J-JY, QC, QZ, and J-CH established the ICH models. J-JY, G-QY, C-XG, and Z-MQ performed the oxylipins quantification and data analysis. W-JZ, H-FS, QH, and RX analyzed and interpreted the results of the RNA-seq experiments. QX and X-YX drafted the manuscript. Q-WY supervised the study. All authors approved the final version of the manuscript.

## Conflict of Interest

The authors declare that the research was conducted in the absence of any commercial or financial relationships that could be construed as a potential conflict of interest.

## Footnotes

1 http://asia.ensembl.org/Rattus_norvegicus/Info/Index

## Supplementary Material

FIGURE S1 Differential production of oxylipin in ICH. **(A)** Violin plots show the increase in oxylipins from DHA (16,17-EpDPE) and LA (13-oxoODE, 9,10-EpOME) in ICH (^∗^ vs Sham group, VIP ≥ 1, *p* < 0.05 using the Mann–Whitney U test and Log_2_FC ≥ 1, *n* = 10); **(B,C)** KEGG analysis of the identified differential oxylipins from AA at 0.5 day **(B)** and 3 days **(C)** after ICH. Red indicates a significant increase in oxylipin; blue indicates that the oxylipin was detected but did not change significantly and white indicates that the oxylipin was not quantitative in the brain.

## Abbreviations

AA: arachidonic acid
AD: Alzheimer’s disease
ALA: α-linolenic acid
ALOX5AP: arachidonate 5-lipoxygenase activating protein
COX: cyclooxygenase
cPLA2: cytosolic phospholipase A2
CYP: cytochrome P450
DEGs: differentially expressed genes
DGLA: dihomo- γ-linolenic acid
DHA: docosahexaenoic acid
DiHETrE: dihydroxyeicosatrienoic acid
EET: epoxy-eicosatrienoic acids
EPA: eicosapentaenoic acid
EpDPE: epoxy-docosapentaenoic acid
EpOME: epoxy-octadecenoic acid
HEPE: hydroxyleicosapentaenoic acid
HETE: hydroxy-eicosatetraenoic acid
ICH: intracerebral hemorrhage
KEGG: Kyoto Encyclopedia of Genes and Genomes
LA: linoleic acid
LC/MS: liquid chromatography tandem mass spectrometry
LOX: lipoxygenase
OPLS-DA: orthogonal projections to latent structures
oxo-ETE: oxo-eicosatetraenoic acid
oxoODE: oxo-octadecadienoic acid
PCA: principal component analysis
PUFAs: polyunsaturated fatty acids
RNA-seq: RNA sequencing
sEH: soluble epoxide hydrolase
VIP: variable importance in projection.
