# Supplementary figures and images for: Quantitative Profiling of Oxylipins in Acute Experimental Intracerebral Hemorrhage
Source: Front Neurosci. 2020 Sep 23;14:777. doi: 10.3389/fnins.2020.00777 (PMC7538633; doi:10.3389/fnins.2020.00777)

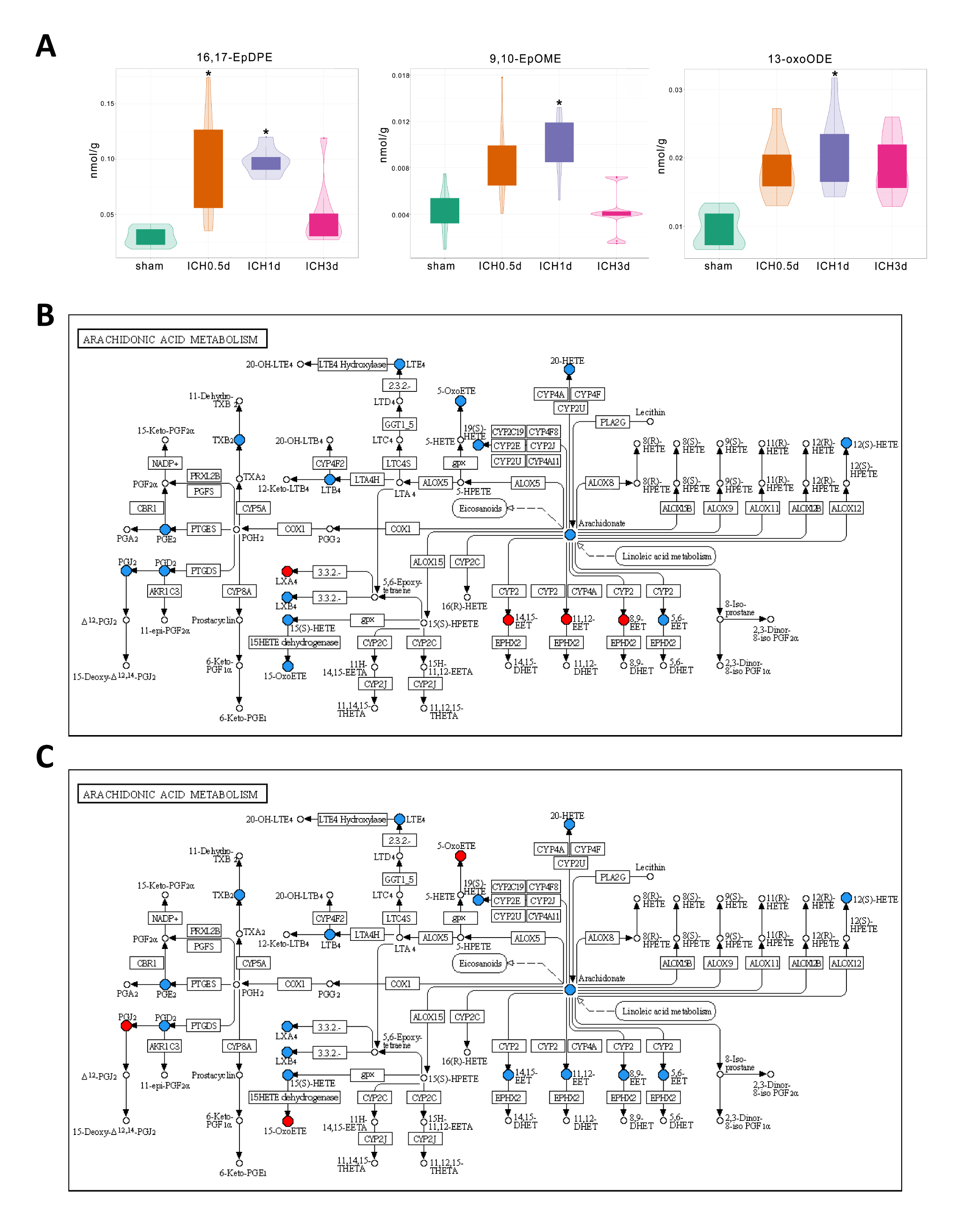

Supplement: FIGURE S1 — Differential production of oxylipin in ICH. (A) Violin plots show the increase in oxylipins from DHA (16,17-EpDPE) and LA (13-oxoODE, 9,10-EpOME) in ICH (∗ vs Sham group, VIP ≥ 1, p < 0.05 using the Mann–Whitney U test and Log2FC ≥ 1, n = 10); (B,C) KEGG analysis of the identified differential oxylipins from AA at 0.5 day (B) and 3 days (C) after ICH. Red indicates a significant increase in oxylipin; blue indicates that the oxylipin was detected but did not change significantly and white indicates that the oxylipin was not quantitative in the brain. [file Image_1.TIF]
