# Supplementary material for: Quantitative Profiling of Oxylipins in Acute Experimental Intracerebral Hemorrhage
Source: Front Neurosci. 2020 Sep 23;14:777. doi: 10.3389/fnins.2020.00777 (PMC7538633; doi:10.3389/fnins.2020.00777)
Supplement: Supplementary file 2 [file Table_1.DOCX]

**Supplementary Table S1** The content of oxylipins in the acute phase of ICH.

| oxylipin | Sham | ICH 0.5d | | | ICH 1d | | | ICH 3d | | |
| --- | --- | --- | --- | --- | --- | --- | --- | --- | --- | --- |
|  | content(nmol/g) | content(nmol/g) | VIP | P-value | content(nmol/g) | VIP | P-value | content(nmol/g) | VIP | P-value |
| **AA** | 603.5±150.8 | 560.9±132.7 | 0.42 | 0.51 | 484.8±152.1 | 0.85 | 0.10 | 575.0±125.1 | 0.31 | 0.65 |
| 14,15-EET | 0.071±0.018 | 0.171±0.063 | 1.50 | 0.00 | 0.204±0.051 | 1.60 | 0.00 | 0.094±0.048 | 0.70 | 0.19 |
| 11,12-EET | 0.054±0.013 | 0.133±0.048 | 1.53 | 0.00 | 0.148±0.035 | 1.59 | 0.00 | 0.070±0.033 | 0.75 | 0.19 |
| 8,9-EET | 0.021±0.007 | 0.062±0.032 | 1.51 | 0.00 | 0.062±0.023 | 1.55 | 0.00 | 0.030±0.013 | 1.11 | 0.08 |
| 5,6-EET | 0.217±0.039 | 0.485±0.411 | 1.01 | 0.07 | 0.288±0.135 | 0.36 | 0.14 | 0.252±0.075 | 0.47 | 0.22 |
| 20-HETE | 0.002±0.001 | 0.004±0.001 | 1.26 | 0.02 | 0.004±0.001 | 1.35 | 0.00 | 0.004±0.002 | 1.23 | 0.02 |
| 5-HETE | 0.789±0.164 | 1.159±0.263 | 1.25 | 0.00 | 1.066±0.257 | 1.01 | 0.01 | 0.76±0.24 | 0.33 | 0.75 |
| 19-HETE | 0.008±0.002 | 0.017±0.004 | 0.73 | 0.00 | 0.015±0.004 | 1.01 | 0.00 | 0.011±0.004 | 1.13 | 0.02 |
| 18-HETE | 0.007±0.002 | 0.010±0.004 | 0.90 | 0.04 | 0.013±0.005 | 1.25 | 0.01 | 0.013±0.004 | 1.64 | 0.00 |
| 17-HETE | 0.001±0.0001 | 0.001±0.0003 | 0.88 | 0.01 | 0.001±0.0005 | 1.04 | 0.04 | 0.001±0.0002 | 1.05 | 0.24 |
| 16-HETE | 0.015±0.006 | 0.025±0.009 | 1.02 | 0.01 | 0.02±0.007 | 0.68 | 0.08 | 0.018±0.007 | 0.49 | 0.29 |
| 15-HETE | 1.322±0.381 | 1.892±0.553 | 0.94 | 0.02 | 1.954±0.492 | 1.05 | 0.01 | 1.661±0.320 | 1.01 | 0.05 |
| 12-HETE | 3.685±2.032 | 6.344±4.513 | 0.99 | 0.11 | 8.10±5.02 | 1.22 | 0.02 | 6.10±3.30 | 1.12 | 0.07 |
| 11-HETE | 0.886±0.268 | 1.177±0.345 | 0.78 | 0.05 | 1.325±0.399 | 1.00 | 0.01 | 1.171±0.19 | 1.16 | 0.01 |
| 9-HETE | 0.179±0.050 | 0.308±0.085 | 1.27 | 0.00 | 0.275±0.068 | 1.10 | 0.00 | 0.218±0.065 | 0.65 | 0.15 |
| PGE2 | 0.841±0.301 | 0.891±0.189 | 0.22 | 0.66 | 1.060±0.189 | 0.58 | 0.11 | 1.123±0.198 | 1.06 | 0.03 |
| PGD2 | 0.759±0.295 | 0.776±0.203 | 0.12 | 0.89 | 0.932±0.203 | 0.49 | 0.19 | 0.996±0.169 | 0.98 | 0.04 |
| 6 keto-PGF1α | 0.227±0.038 | 0.268±0.076 | 0.46 | 0.15 | 0.344±0.076 | 1.06 | 0.02 | 0.271±0.041 | 1.13 | 0.02 |
| PGF2α | 0.680±0.221 | 0.446±0.073 | 1.22 | 0.01 | 0.552±0.123 | 0.63 | 0.13 | 0.544±0.094 | 0.77 | 0.10 |
| PGJ2 | 1.159±0.458 | 2.257±1.039 | 1.01 | 0.01 | 3.033±1.343 | 1.31 | 0.00 | 3.845±1.086 | 1.91 | 0.00 |
| LTE4 | 0.022±0.011 | 0.104±0.049 | 0.97 | N/A | 0.155±0.089 | 1.41 | N/A | 0.053±0.069 | 0.32 | N/A |
| LTB4 | 0.004±0.001 | 0.008±0.003 | 1.35 | 0.01 | 0.006±0.002 | 0.72 | 0.15 | 0.005±0.003 | 0.59 | 0.28 |
| TXB2 | 0.243±0.085 | 0.192±0.030 | 0.82 | 0.10 | 0.277±0.069 | 0.27 | 0.34 | 0.265±0.057 | 0.41 | 0.51 |
| LipoxinA4 | 0.003±0.001 | 0.006±0.003 | 1.11 | 0.00 | 0.004±0.002 | 0.74 | 0.04 | 0.003±0.001 | 0.04 | 0.85 |
| 5,6-DiHETrE | 0.004±0.002 | 0.010±0.005 | 1.44 | 0.00 | 0.013±0.005 | 1.46 | 0.00 | 0.006±0.003 | 0.99 | 0.03 |
| 5-iso PGF2VI | 0.037±0.012 | 0.046±0.014 | 0.64 | 0.13 | 0.041±0.012 | 0.37 | 0.43 | 0.036±0.010 | 0.06 | 0.81 |
| 5-oxoETE | 0.101±0.024 | 0.184±0.033 | 1.53 | 0.00 | 0.140±0.044 | 0.86 | 0.03 | 0.209±0.078 | 1.68 | 0.00 |
| 15-oxoETE | 0.048±0.011 | 0.096±0.033 | 1.30 | 0.00 | 0.071±0.035 | 0.62 | 0.07 | 0.148±0.061 | 1.73 | 0.00 |
| **Linoleic Acid** | 45.31±16.67 | 40.64±25.88 | 0.33 | 0.64 | 27.8±15.4 | 0.95 | 0.03 | 34.3±12.9 | 0.64 | 0.12 |
| 9-oxoODE | 0.008±0.005 | 0.011±0.008 | 0.38 | 0.33 | 0.007±0.004 | 0.07 | 0.65 | 0.014±0.008 | 0.64 | 0.07 |
| 13-oxoODE | 0.010±0.003 | 0.018±0.004 | 1.48 | 0.00 | 0.020±0.005 | 1.40 | 0.00 | 0.019±0.004 | 1.72 | 0.00 |
| 9,10-DiHOME | 0.007±0.002 | 0.013±0.004 | 1.46 | 0.00 | 0.015±0.003 | 1.53 | 0.00 | 0.009±0.002 | 0.96 | 0.09 |
| 9,10-EpOME | 0.004±0.002 | 0.009±0.003 | 1.16 | 0.00 | 0.010±0.003 | 1.23 | 0.00 | 0.004±0.002 | 0.01 | 0.84 |
| 12,13-EpOME | 0.005±0.002 | 0.006±0.004 | 0.45 | 0.27 | 0.006±0.002 | 0.55 | 0.11 | 0.004±0.002 | 0.24 | 0.87 |
| **EPA** | 7.205±1.912 | 10.03±1.66 | 1.19 | 0.00 | 9.861±1.745 | 1.10 | 0.00 | 10.02±1.69 | 1.43 | 0.00 |
| 18-HEPE | 0.004±0.001 | 0.012±0.006 | 1.00 | 0.01 | 0.012±0.005 | 1.24 | 0.00 | 0.010±0.005 | 1.62 | 0.00 |
| 15-HEPE | 0.0030 | 0.005±0.002 | 1.20 | N/A | 0.005±0.001 | 1.57 | N/A | 0.004±0.002 | 2.03 | N/A |
| 12-HEPE | 0.079±0.061 | 0.256±0.303 | 1.20 | 0.10 | 0.308±0.290 | 1.37 | 0.04 | 0.236±0.193 | 1.39 | 0.03 |
| 5-HEPE | 0.005±0.001 | 0.011±0.003 | 1.67 | 0.00 | 0.012±0.002 | 1.64 | 0.00 | 0.008±0.002 | 1.69 | 0.00 |
| RvD5 | 0.002±0.0005 | 0.003±0.0005 | 0.85 | 0.05 | 0.003±0.006 | 0.83 | 0.03 | 0.002±0.0006 | 0.85 | 0.22 |
| LXA5 | 0.012±0.004 | 0.014±0.003 | 0.76 | 0.25 | 0.020±0.004 | 0.91 | 0.00 | 0.012±0.007 | 0.02 | 0.89 |
| 5(S),15(S)-DiHETE | 0.025±0.005 | 0.034±0.007 | 1.10 | 0.01 | 0.033±0.012 | 0.81 | 0.07 | 0.019±0.008 | 1.07 | 0.04 |
| 5,6-DiHETE | N/A | 0.048±0.014 | 1.01 | N/A | 0.061±0.020 | 0.97 | N/A | 0.025±0.011 | 1.46 | N/A |
| **DHA** | 1650.0±388.4 | 1425.5±420.2 | 0.59 | 0.23 | 1149.6±235.3 | 1.19 | 0.00 | 1342.6±364.5 | 0.86 | 0.08 |
| 4-HDHA | 12.329±5.033 | 16.17±7.10 | 0.55 | 0.18 | 12.93±3.693 | 0.09 | 0.77 | 14.28±4.91 | 0.48 | 0.39 |
| 7-HDHA | 2.532±0.942 | 3.225±1.209 | 0.50 | 0.17 | 2.601±0.802 | 0.04 | 0.86 | 2.703±1.026 | 0.17 | 0.70 |
| 14-HDHA | 14.735±9.148 | 17.24±8.92 | 0.56 | 0.54 | 15.57±9.27 | 0.21 | 0.84 | 21.96±10.46 | 1.06 | 0.12 |
| 17-HDHA | 5.756±2.720 | 6.916±3.418 | 0.34 | 0.41 | 5.50±1.64 | 0.05 | 0.80 | 6.774±2.799 | 0.49 | 0.42 |
| PDX | 0.094±0.062 | 0.102±0.070 | 0.12 | 0.79 | 0.065±0.018 | 0.38 | 0.17 | 0.092±0.039 | 0.30 | 0.93 |
| 16,17-EpDPE | 0.030±0.008 | 0.092±0.046 | 1.48 | 0.00 | 0.098±0.012 | 1.63 | 0.00 | 0.048±0.028 | 1.06 | 0.07 |
| **α-Linolenic Acid** | 0.599±0.450 | 0.936±0.220 | 1.19 | 0.05 | 0.943±0.301 | 1.10 | 0.06 | 0.540±0.137 | 0.03 | 0.70 |
| 9-HOTrE | 0.001±0.0003 | 0.002±0.001 | 0.78 | 0.08 | 0.002±0.001 | 0.43 | 0.19 | 0.001±0.0003 | 0.58 | 0.33 |
| **γ-Linolenic Acid** | 1.176±0.315 | 1.532±0.427 | 0.89 | 0.05 | 1.471±0.308 | 0.98 | 0.05 | 0.993±0.264 | 0.68 | 0.18 |
| 13-HOTrE | 0.003±0.001 | 0.004±0.003 | 0.96 | 0.25 | 0.002±0.002 | 0.84 | 0.61 | 0.002±0.001 | 0.44 | 0.34 |
| **Dihomo-γ-Linolenic Acid** | 119.7±28.8 | 106.2±28.7 | 0.37 | 0.30 | 98.1±22.1 | 0.78 | 0.07 | 99.8±27.0 | 0.79 | 0.12 |
| PGE1 | 0.006±0.002 | 0.005±0.003 | 0.05 | 0.36 | 0.007±0.003 | 0.40 | 0.79 | 0.007±0.002 | 0.44 | 0.45 |
| PGD1 | 0.025±0.004 | 0.026±0.006 | 0.26 | 0.53 | 0.028±0.006 | 0.32 | 0.32 | 0.034±0.006 | 1.52 | 0.00 |
| PGF1α | 0.030±0.008 | 0.020±0.004 | 1.26 | 0.00 | 0.027±0.007 | 0.35 | 0.42 | 0.025±0.005 | 0.85 | 0.08 |
| 5-HETrE | 0.004±0.001 | 0.007±0.003 | 1.06 | 0.02 | 0.008±0.002 | 1.32 | 0.00 | 0.005±0.003 | 0.17 | 0.51 |

Data present mean ± SD

VIP values were calculated by an OPLS-DA model using the metaboanalyst software.

P values were calculated by Mann-Whitney test, compared to the sham group.
